# Effect of supplement with lactic-acid producing bacteria on fatigue and physical activity in patients with chronic fatigue syndrome

**DOI:** 10.1186/1475-2891-8-4

**Published:** 2009-01-26

**Authors:** Åsa Sullivan, Carl E Nord, Birgitta Evengård

**Affiliations:** 1 Division of Clinical Microbiology, F82, Department of Laboratory Medicine, Karolinska University Hospital Huddinge, Karolinska Institutet, SE-141 86 Stockholm, Sweden; 2 Division of Infectious Diseases, Department of Clinical Microbiology, Umeå University, Umeå, Sweden

## Abstract

Disturbances in intestinal microbial ecology and in the immune system of the host have been implicated as a part of the pathogenesis in chronic fatigue syndrome. Probiotic lactic acid producing bacteria have been shown to prevent and alleviate gastrointestinal disturbances and to normalize the cytokine profile which might be of an advantage for patients suffering from chronic fatigue syndrome. The aim of the study was to evaluate the effect of *Lactobacillus paracasei *ssp. *paracasei *F19, *Lactobacillus acidophilus *NCFB 1748 and *Bifidobacterium lactis *Bb12 on fatigue and physical activity in CFS patients. Fifteen patients fulfilling the criteria set by international researchers in the field at the US Centre for Disease Control and Prevention in 1994 for chronic fatigue syndrome, were included in the study. The patients had high fatigue severity scores and high disability scores. During the first two weeks baseline observations without treatment were assessed, succeeded by four weeks of intake of a probiotic product and a four-week follow-up period. The fatigue, health and physical activity was assessed by the use of the Visual Analogue Scales and the SF-12 Health Survey. Faecal samples were collected and the normal microflora was analysed. Neurocognitive functions improved during the study period while there were no significant changes in fatigue and physical activity scores. No major changes occurred in the gastrointestinal microflora. At the end of the study 6 of 15 patients reported that they had improved according to the assessment described. The findings in this study that improvement of health is possible to achieve should encourage further studies with interventions with probiotics in patients with CFS.

## Background

Chronic fatigue syndrome (CFS) is a medically unexplained illness characterized by persistent and debilitating fatigue with a minimum duration of six months. The current case definition set by an international expert group 1994 includes at least 4 of 8 designated symptoms [1,2]. The prevalence of CFS range between 0.2 and 2.6% in patients attending primary care and in community based surveys [3]. In a recent nationwide study from Sweden the prevalence of CFS-like illness (defined as meeting all self-reported criteria for CFS but lacking the physical examination) was 2.4% [4]. The aetiology and pathophysiology of the syndrome remains unknown but several factors have been discussed. Among factors that have been implicated in triggering or mediating the course of the disease are infective disorders, immune system disorders, neuroendocrine abnormalities and neuropsychological impairment [5,6]. It has been suggested that immune dysfunctions that has been observed in CFS patients account for a number of the described symptoms [7]. The CFS patients have a cytokine imbalance in the peripheral blood compartment and the system is biased towards a T-helper (Th) 2 type immunity-oriented pattern. This is reflected in the high incidence of allergies in CFS patients [8]. Furthermore, comorbidity between CFS and irritable bowel syndrome (IBS) has been identified in a number of studies and a high degree of overlapping symptoms has been reported [9]. The irritable bowel syndrome is a functional bowel disorder characterized by symptoms of abdominal pain or discomfort that is associated with disturbed defecation [10]. There is evidence suggesting that the intestinal microflora of patients with IBS differs from that of healthy individuals and that the patients have an abnormal fermentation of food residues [11]. The intestinal microflora in CFS patients have also been shown to be altered with low levels of *Escherichia coli *and *Bifidobacterium *species and with significant increased numbers of enterococci compared to healthy controls [12]. An elevated number of Candida albicans in the faecal microflora of CFS patients during the acute phase of illness has recently been reported [13]. Recently it has also been pointed out that oxidative stress and food intolerance might be involved in the pathogenesis of CFS and in the symptom presentation [14]. It is still uncertain whether oxidative stress is a cause or a result of the disease. However, various antioxidants have shown promise as a part of CFS treatment protocols. The information concerning food intolerance and CFS is limited but there are indications of alleviation of symptoms and reduction in fatigue when food elimination protocols have been followed [14].

There is increasing evidence that consumption of foods containing microorganisms is beneficial in prevention and treatment of gastrointestinal disorders [15]. Several trials have been performed on the effect of probiotics in IBS [16-20]. The administration of probiotics resulted in reduced flatulence and abdominal pain in three of the studies. However, in one study where enterocoated tablets were used and in one where a lower concentration of probiotic lactobacilli were administered, no effect was observed [19,20].

Strains of lactobacilli and bifidobacteria have been investigated for their antioxidative activity. Several species have been identified that are capable of chelating metal ions, scavenging reactive oxygen species and that possess reducing activity [21]. Human intestinal isolates of lactobacilli have also been shown to be strong stimulators of interleukin 12 (IL-12) from monocytes [22]. It has been suggested that supplements with lactic acid producing bacteria apart from modifying the microflora also deviate the immune phenotype and correct the Th2-type bias that promotes allergy [23]. Probiotic lactobacilli have further been shown to be beneficial in the treatment of food allergy [24]. Lactic acid producing bacteria have recently been suggested as therapeutic agents in the treatment of CFS [25]. Potential benefits for CFS patients could be regulation of the composition of the microflora, the impact on the cytokine balance and the action of the probiotic strains as antioxidants. There are no studies performed on the effects of probiotics in CFS.

The aim of the study was to evaluate the effect of *Lactobacillus paracasei *ssp. *paracasei *F19, *Lactobacillus acidophilus *NCFB 1748 and *Bifidobacterium lactis *Bb12 on fatigue and physical activity in CFS patients.

## Methods

### Patients

Fifteen patients (10 females and 5 men) fulfilling the 1994 CDC criteria for chronic fatigue syndrome were included in the study. The patients had both high fatigue severity scores and high disability scores. The mean age was 43 years (range 30–56 years, 45 years for women and 39 years for men). None of the patients had used antimicrobial agents within the preceding three months. The patients did not suffer from lactose intolerance or underlying chronic inflammatory diseases. The study was approved by the regional ethical committee in Stockholm (Ref 2005/888-31/1).

### Trial design

The study was designed as an open pilot study with a total duration of ten weeks per participant. During the first two weeks baseline observations without treatment were assessed (to provide baseline mean values of faecal microflora and calprotecin levels) succeeded by four weeks of treatment and a four-week follow-up period

### Administration of supplement

The probiotic product was administered twice daily (2 dl × 2) during 30 days with start on day 14. The product contained 10^8 ^colony forming units (cfu)/ml of the strains *Lactobacillus *F19, *L. acidophilus *NCFB 1748 and *B. lactis *Bb12 (Cultura Dofilus Natural Yogurt, Arla Foods, Stockholm, Sweden). The fat content was 1.5%.

### Fatigue and physical activity

At baseline (day 0), at the end of treatment (day 42) and at the end of the study (day 70) the patients were assessed by the Visual Analogue Scales (VAS) [26] to measure the intensity of symptoms (fatigue, muscle and neurocognitive symptoms as subjectively reported affecting short term memory and capacity to concentrate). The patients' self-reported physical activity level and general health status were measured with the SF-12 Health Survey once weekly during the study period [27].

At the end of the study the patients were asked to evaluate the treatment by the use of a four-point scale; complete recovery, improvement, unchanged or impairment of symptoms.

### Sampling procedures – faecel samples

Stool specimens were collected before administration of the probiotic product (days 0 and 14), on the last day of administration (day 42) and after the administration (day 70). The faecal samples were collected in sterile plastic jars and were frozen at -70°C until assayed.

### Faecal concentrations of calprotectin

Parts of the collected stool samples were used for determinations of faecal concentrations of calprotectin. Faecal calprotectin has been shown to closely correlate with colonic macroscopic and histological inflammation. The stool samples were prepared and analysed according to the manufacturers instructions (Calprest Eurospital SpA, Trieste, Italy). The samples were suspended in buffer, homogenized and centrifuged followed by ELISA assays of the supernatants [28].

### Microbiological procedures

The stool specimens were suspended in pre-reduced peptone-yeast extract medium, diluted to 10^-7 ^and inoculated on non-selective and selective media. The aerobic agar plates were incubated for 24 hours at 37°C and the anaerobic plates for 48 hours at 37°C in anaerobic jars (GasPak; BBL, Cockeysville, MD, USA). After incubation different colony types were counted, isolated in pure cultures and identified to genus level. All isolates were analysed according to Gram-reaction and colony morphology, followed by biochemical tests. API 20E test kit (Biomeriux SA, Marcy l'Etoile, France) was used for identification of Enterobacteriaceae. Anaerobic microorganisms were identified by gas-liquid chromatography of metabolites from glucose. The lower limit of detection was 10^2 ^microorganisms per gram faeces [29].

### Compliance

Colony forming units of lactobacilli exhibiting Gram-stain morphology similar to *Lactobacillus *F19 were examined by randomly amplified polymorphic DNA (RAPD-PCR) to verify the identification. The primer Lbc-19 (5'-AGTAGCCAC3') was used for screening and OPA-02 (5'-TGCCGAGCTC-3') for the identification [30]. This was performed to control for compliance.

### Statistical analyses

The quantitative alterations in the normal microflora and the levels of calprotectin were compared within the group between pre-treatment (mean of days 0 and 14) and the end of treatment (day 42) and between pre-treatment and four weeks after treatment (day 70) and were analysed by Wilcoxon's signed rank test for paired samples. *P *values = 0.05 were considered statistically significant and were adjusted for multiple analyses.

## Results

### Fatigue

When VAS mean numbers were analysed a significant difference was found for neurocognitive functions which improved (p = 0,040) from measurement before treatment (baseline) and at end of follow-up (day 70). No gender-difference was observed. If data were analysed on an individual level differences were found as shown in Table 1. Both women and men reported both improvement and worsening of fatigue, muscle-symptoms and of neurocognitive function.

**Table 1:** Change of measurements on the visual analogue scale 0–10 (VAS) from baseline (day 0) to follow-up (day 70).

Change of VAS	Women, no			Men, no		
	
	Fatigue	Muscle	Neurocogn	Fatigue	Muscle	Neurocogn
> 2	1	0	2	0	1	0

0.5–2	5	2	1	1	0	2

0	1	3	4	2	1	2

-(0.5–2)	1	4	3	1	1	0

< -2	2	1	0	1	2	1

Fatigue = general fatigue, Muscle = muscle symptoms, Neurocogn = neurocognitive symptoms as short-term memory and capacity to concentrate

### Health and physical activity measured by SF-12

A clear reduction of levels of health were reported at baseline (day 0) for both measurements (physical and mental) when compared to the norms for the general Swedish population (Table 2).

**Table 2:** SF-12 measurements of physical and mental health at baseline (day 0) with norm for the general Swedish population by age-group in parenthesis.

	Physical health	Mental health
Total population	31.0 (51.3)	44.7 (53.0)

Women	30.2 (50.6)	48.2 (52.6)

Men	32.6 (52.9)	37.7 (52.8)

There was no significant change during the treatment period and during the follow-up.

On an individual basis there were differences as some subjects reported improvement in one or both types of health while others noticed no change or worsening of health.

Four of 10 women reported improvement in physical health and 2 in mental health at day 70. One of 5 men reported improvement in physical health and 1 in mental health as measured by SF-12 (Table 3).

**Table 3:** Number of women and men reporting change in measurement in SF-12 from baseline (day 0) to end of follow-up (day 70).

Change of measurement in SF-12	Women, No		Men, No	
		
	Physical	Mental	Physical	Mental
		
> 10	1	2	0	1
		
5–8	0	0	1	0
		
1–4	3	0	0	0
		
0	4	5	3	1
		
-1–4	1	0	0	0
		
-5–8	0	0	1	0
		
< -10	1	3	0	3

### Subjects report of response

Six of 15 patients reported that they improved when asked for four given alternatives. (Worsening- one female, no improvement- five women and three men, improvement- four women and two men, and complete recovery- none).

### Faecal concentrations of calprotectin

The faecal calprotectin levels were normal in all patients (< 50 mg/kg faeces) although 3 patients had occasionally higher values (one sample per patient on days 14, 42 and 70 respectively).

### Gastrointestinal microflora

No statistically significant alterations in the aerobic intestinal microflora were found while there was increased numbers of lactobacilli on day 42 and of veillonella on day 70. The probiotic strain *Lactobacillus *F19 was recovered from all patients on day 42.

## Discussion

Due to the complexity of the cause of CFS it is not reasonable to believe that a pharmacological compound can be found that will cure all patients [5]. Also, the high impact on society points at the necessity to find treatments through other mechanisms [31]. As the bowel is an important organ for equilibrium in health [25] the use of probiotics as "anti-fatigue-food" is a temptating treatment both from the point of cost and availability as from biological functions as the neurocognitive system can be influenced through immune reactions involving cytokines and anti-oxidants. The theoretical background is thus solid enough to justify a performance of an intervention. Disturbances reported in the gut flora could not be reproduced in this study. The design included a period of baseline observation, treatment and a follow-up period. The patients became their own controls. The advantage here is that confounding factors that exist between individuals affecting the flora as diet and other daily behaviours are controlled for. However, as CFS has a fluctuating natural course it could affect the outcome. By including a satisfying large number of patients this could be controlled for. The present study is a pilot study.

Another comment that is frequently made is that the placebo effect should be high in this condition as it is of subjective nature. However, it has been demonstrated that the placebo effects in CFS is low [32].

The different responses in patients demonstrate the heterogeneity of the patient population. Recent studies have pointed out the need for subgrouping of patients or maybe even a new definition of the syndrome [2,4,33]. When the average is calculated there is no influence of the intervention but for individuals it can have a clear effect whether it is improvement or worsening. The low number of subjects makes it impossible to study gender differences. There is a significant improvement for the studied population for neurocognitive functions with sustainability as it is demonstrated at the end of the follow-up (day 70). Also almost half of the subjects report improvement on the four-item scale and a number of subjects report improvement in general health according to SF-12.

What is very clear is the severity of the condition as described through SF12 for both physical and mental health. For both women and men their report of physical and mental health is less than the norm for the general Swedish population age 75 years and above. Studies from the US show that CFS can be as disabling as multiple sclerosis, lupus, rheumatoid arthritis, heart disease, end-stage renal disease, chronic obstructive pulmonary disease and similar chronic conditions.

The high cost for society has been calculated by the Center for Disease Control, Atlanta to be more than 9 billion dollars in the US in lost productivity [31], not including medical costs or disability benefits. Our findings confirm that not only is this a costly condition for society but the impairment of reported physical and mental health for the individual is severe and a condition that should be taken seriously by the medical profession.

## Conclusion

The findings in this study that improvement of health is possible to achieve should encourage further studies with interventions with probiotics in patients with CFS.

The challenge for the future is to identify the responders to the therapy with probiotics as this pilot study demonstrates that some individuals do respond with less fatigue, less bodily symptoms and better neurocognitive functions.

## Competing interests

The authors declare that they have no competing interests.

## Authors' contributions

AS contributed in the preparation of study protocols, data collection, analyses of the intestinal microflora and the statistical analyses, CEN participated in the design of the study and BE took part in the preparations and the analyses of the fatigue and health scales. All three authors participated in the manuscript preparation and have read and approved the final version.

## References

[B1] Fukuda K, Straus SE, Hickie I, Sharpe MC, Dobbins JG, Komaroff A (1994). The chronic fatigue syndrome: a comprehensive approach to its definition and study. International Chronic Fatigue Syndrome Study Group. Ann Intern Med.

[B2] Reeves WC, Lloyd A, Vernon SD, Klimas N, Jason LA, Bleijenberg G, Evengard B, White PD, Nisenbaum R, Unger ER (2003). Identification of ambiguities in the 1994 chronic fatigue syndrome research case definition and recommendations for resolution. BMC Health Serv Res.

[B3] Ranjith G (2005). Epidemiology of chronic fatigue syndrome. Occup Med (Lond).

[B4] Evengard B, Jacks A, Pedersen NL, Sullivan PF (2005). The epidemiology of chronic fatigue in the Swedish twin registry. Psychological Medicine.

[B5] Evengard B, Klimas N (2002). Chronic fatigue syndrome: probable pathogenesis and possible treatments. Drugs.

[B6] Adler RH (2004). Chronic fatigue syndrome (cfs). Swiss Med Wkly.

[B7] Patarca R (2001). Cytokines and chronic fatigue syndrome. Ann N Y Acad Sci.

[B8] Friedberg F, Dechene L, McKenzie MJ, Fontanetta R (2000). Symptom patterns in long-duration chronic fatigue syndrome. J Psychosom Res.

[B9] Whitehead WE, Palsson O, Jones KR (2002). Systematic review of the comorbidity of irritable bowel syndrome with other disorders: what are the causes and implications?. Gastroenterology.

[B10] Drossman DA, Camilleri M, Mayer EA, Whitehead WE (2002). AGA technical review on irritable bowel syndrome. Gastroenterology.

[B11] Madden JA, Hunter JO (2002). A review of the role of the gut microflora in irritable bowel syndrome and the effects of probiotics. Br J Nutr.

[B12] Butt HL, Dunstan RH, McGregor NR, Roberts TK 'Bacterial colonosis' in patients with persistent fatigue. Proceedings of the AHMF International Clinical and Scientific Conference; Sydney, Australia.

[B13] Evengård B, Gräns H, Wahlund E, Nord CE (2007). Elevated levels of Candida albicans in the faecal microflora of chronic fatigue syndrome patients during the acute phase of illness. Scand J Gastroent.

[B14] Logan AC, Wong C (2001). Chronic fatigue syndrome: oxidative stress and dietary modifications. Altern Med Rev.

[B15] Sullivan A, Nord CE (2005). Probiotics and gastrointestinal diseases. J Intern Med.

[B16] Halpern GM, Prindiville T, Blankenburg M, Hsia T, Gershwin ME (1996). Treatment of irritable bowel syndrome with Lacteol Fort: a randomized, double-blind, cross-over trial. Am J Gastroenterol.

[B17] Niedzielin K, Kordecki H, Birkenfeld B (2001). A controlled, double-blind, randomized study on the efficacy of Lactobacillus plantarum 299V in patients with irritable bowel syndrome. Eur J Gastroenterol Hepatol.

[B18] Nobaek S, Johansson ML, Molin G, Ahrne S, Jeppsson B (2000). Alteration of intestinal microflora is associated with reduction in abdominal bloating and pain in patients with irritable bowel syndrome. Am J Gastroenterol.

[B19] Sen S, Mullan MM, Parker TJ, Woolner JT, Tarry SA, Hunter JO (2002). Effect of Lactobacillus plantarum 299v on colonic fermentation and symptoms of irritable bowel syndrome. Dig Dis Sci.

[B20] MA O'Sullivan, CA O'Morain (2000). Bacterial supplementation in the irritable bowel syndrome. A randomised double-blind placebo-controlled crossover study. Dig Liver Dis.

[B21] Lin MY, Yen CL (1999). Antioxidative ability of lactic acid bacteria. J Agric Food Chem.

[B22] Hessle C, Hanson LA, Wold AE (1999). Lactobacilli from human gastrointestinal mucosa are strong stimulators of IL-12 production. Clin Exp Immunol.

[B23] Cross ML, Gill HS (2001). Can immunoregulatory lactic acid bacteria be used as dietary supplements to limit allergies?. Int Arch Allergy Immunol.

[B24] Majamaa H, Isolauri E (1997). Probiotics: a novel approach in the management of food allergy. J Allergy Clin Immunol.

[B25] Logan AC, Venket Rao A, Irani D (2003). Chronic fatigue syndrome: lactic acid bacteria may be of therapeutic value. Med Hypotheses.

[B26] Collins SL, Moore RA, McQuay HJ (1997). The visual analogue pain intensity scale: what is moderate pain in millimetres?. Pain.

[B27] Ware JE, Sherbourne CD (1992). The MOS 36-item short-form health survey (SF-36). I. Conceptual framework and item selection. Med Care.

[B28] Fagerberg UL, Loof L, Merzoug RD, Hansson LO, Finkel Y (2003). Fecal calprotectin levels in healthy children studied with an improved assay. J Pediatr Gastroenterol Nutr.

[B29] Sullivan Å, Johansson A, Svenungsson B, Nord CE (2004). Effect of lactobacillus F19 on the emergence of antibiotic-resistant microorganisms in the intestinal microflora. J Antimicrob Chemother.

[B30] Björneholm S, Eklöw A, Saarela M, Mättö J (2002). Enumeration and identification of *Lactobacillus paracasei *subsp. *paracasei *F19. Microbial Ecology in Health and Disease.

[B31] Reynolds KJ, Vernon SD, Bouchery E, Reeves WC (2004). The economic impact of chronic fatigue syndrome. Cost Eff Resour Alloc.

[B32] Cho HJ, Hotopf M, Wessely S (2005). The placebo response in the treatment of chronic fatigue syndrome: a systematic review and meta-analysis. Psychosom Med.

[B33] Jason LA, Corradi K, Torres-Harding S, Taylor RR, King C (2005). Chronic fatigue syndrome: the need for subtypes. Neuropsychol Rev.

